# Cumulative COVID-19 Disparities in Nursing Home: Focusing on Geographical Factors and Racial Components

**DOI:** 10.1093/geroni/igab046.3144

**Published:** 2021-12-17

**Authors:** Sungjae Hong, Shannon Meija

**Affiliations:** 1 University of Illinois--Urbana-Champaign, Champaign, Illinois, United States; 2 University of Illinois, Champaign, Illinois, United States

## Abstract

The impact of COVID-19 has been greatest in vulnerable US populations. This study examines the cumulative geographical and racial disparities of COVID-19 cases in nursing homes. Analysis of COVID-19 Nursing Home Data from Centers for Medicare & Medicaid Services was limited to weekly reports from the nursing homes that reported the ratio of black residents, from 2020-05-31 to 2021-01-17 (N=268,222 from 8,026 nursing homes). The outcomes were weekly COVID-19 cases and death per 1,000 occupied beds. Nursing homes were categorized by a geographic (rural vs. urban) and racial composition (>50% of residents are black vs. else). Elapsed time and county-level weekly COVID-19 cases and deaths/1,000 people were the key covariates. Multilevel zero-inflated negative binomial regression revealed evidence of cumulative COVID-19 disparity between rural and urban nursing homes. At the earliest time, COVID-19 incidence was lower in rural nursing homes than in urban nursing homes (IRR=0.406 for cases, 0.034 for death). The significant interaction with time implied that, over and above evolving disease prevalence, rural nursing homes became more likely than urban nursing homes to experience COVID-19 over time (IRR=1.057 for cases, 1.193 for death). Nursing homes, with >50% black residents, were more likely to experience COVID-19 than their counterparts at the earliest time (IRR=1.339 for cases, 5.630 for death), but independent of local disease prevalence, this disparity decreased over time (IRR=0.973 for cases, 0.972 for death). Our findings suggest that racial and geographic factors contribute to the cumulation of disadvantage during the COVID-19 crisis at the second half of 2020.

